# *TERT* promoter wild-type glioblastomas show distinct clinical features and frequent PI3K pathway mutations

**DOI:** 10.1186/s40478-018-0613-2

**Published:** 2018-10-17

**Authors:** Erik A. Williams, Julie J. Miller, Shilpa S. Tummala, Tristan Penson, A. John Iafrate, Tareq A. Juratli, Daniel P. Cahill

**Affiliations:** 1 Translational Neuro-Oncology Laboratory, Department of Neurosurgery, Massachusetts General Hospital Cancer Center, Harvard Medical School, 55 Fruit street, Boston, MA USA; 2 Department of Pathology, Massachusetts General Hospital, Harvard Medical School, Boston, MA USA; 3 Stephen E. and Catherine Pappas Center for Neuro-Oncology, Department of Neurology, Massachusetts General Hospital, Harvard Medical School, 55 Fruit Street, Boston, MA 02114 USA

**Keywords:** Glioma, PI3K pathway, TERT promoter, IDH1, H3F3A, BAF complex, Cerebellum

## Abstract

*TERT* promoter (*TERT*p) mutations are found in the majority of World Health Organization (WHO) grade IV adult *IDH* wild-type glioblastoma (*IDH*-wt GBM). Here, we characterized the subset of *IDH*-wt GBMs that do not have *TERT*p mutations. In a cohort of 121 adult grade IV gliomas, we identified 109 *IDH*-wt GBMs, after excluding 11 *IDH*-mutant cases and one *H3F3A -*mutant case. Within the *IDH*-wt cases, 16 cases (14.7%) were *TERT*p wild-type (*TERT*p-wt). None of the 16 had *BRAF* V600E or *H3F3A* G34 hotspot mutations. When compared to TERTp mutants, patients with *TERT*p-wt GBMs, were significantly younger at first diagnosis (53.2 years vs. 60.7 years, *p* = 0.0096), and were more frequently found to have cerebellar location (*p* = 0.0027). Notably, 9 of 16 (56%) of *TERT*p-wt GBMs contained a *PIK3CA* or *PIK3R1* mutation, while only 16/93 (17%) of *TERT*p-mutant GBMs harbored these alterations (*p* = 0.0018). As expected, 8/16 (50%) of *TERT*p-wt GBMs harbored mutations in the BAF complex gene family (*ATRX, SMARCA4*, *SMARCB1*, and *ARID1A*), compared with only 8/93 (9%) of *TERT*p-mutant GBMs (*p* = 0.0003). Mutations in BAF complex and PI3K pathway genes co-occurred more frequently in *TERT*p-wt GBMs (*p* = 0.0002), an association that has been observed in other cancers, suggesting a functional interaction indicative of a distinct pathway of gliomagenesis. Overall, our finding highlights heterogeneity within WHO-defined *IDH* wild-type GBMs and enrichment of the *TERT*p-wt subset for BAF/PI3K-altered tumors, potentially comprising a distinct clinical subtype of gliomas.

**Electronic supplementary material:**

The online version of this article (10.1186/s40478-018-0613-2) contains supplementary material, which is available to authorized users.

## Introduction

Glioblastoma (GBM) is the most frequent and deadly primary brain tumor, accounting for approximately 45–50% of all primary malignant brain tumors [17, 18]. GBM is a heterogeneous entity, with a wide mutational spectrum. There has been an ever-increasing focus on molecular classification in GBM, to develop insights into the biology of this tumor and to subsequently improve diagnosis and treatment.

To emphasize the importance of molecular markers, the 2016 World Health Organization (WHO) revised neuropathological criteria identifies three categories of grade IV diffuse glioma. Two categories of GBM arise based on clustered genetic alterations, histologic variants, and clinical data [15], *IDH* wild-type and *IDH* mutant. An additional category of *H3F3A* K27 M mutant midline glioma has been designated grade IV, due the often poor prognosis of patients with these tumors. While *IDH* and *H3F3A* mutations identify gliomas with a distinct molecular origin, the remaining *IDH* wild-type subgroup of GBM, as it is defined currently, still contains significant heterogeneity. Emerging evidence indicates that *TERT* promoter (*TERT*p) mutations, which are common in these tumors, could additionally be useful clinically to classify *IDH* wild-type GBMs into subgroups with specific clinical courses [7, 12].

Here, we evaluated *TERT*p wild-type (*TERT*p-wt) GBMs to compare them to their *TERT*p mutant counterpart GBMs. We performed sequencing on a broad panel of genes and evaluated for the presence of fusions in a cohort of GBMs, to evaluate the mutational profile of *TERT*p-wt GBMs. In addition, we examined the clinical characteristics of this group.

## Material and methods

The study was reviewed and approved by the human subjects’ institutional review boards of the Dana-Farber Cancer Institute and Massachusetts General Hospital (P10–454) and complied with HIPAA guidelines. We retrospectively reviewed the genomic database at our institution for adult GBM cases submitted for genotyping using the SNaPshot panel version 2. Demographic, treatment and follow-up data were retrospectively collected.

### SNaPshot next generation sequencing archer® FusionPlex®

Specimens were subjected to genomic analysis utilizing SNaPshot^6^, a hybrid capture based method for single nucleotide variant (SNV) and insertion/deletion (indel) detection in tumor DNA. SNaPshot targets 108 genetic loci frequently mutated in 15 cancer genes, including *TERT* promoter, *IDH1*/*2*, *TP53*, *ATRX, PIK3CA, PIK3R1, NF1*, and *STAG2*. The detailed list of all genes included in the SNaPshot v2 panel is shown in Additional file 1.

### Archer® FusionPlex®

Extracted tumor RNA were interrogated for fusions by the Archer® FusionPlex® Solid Tumor (AK0034) kit [25]. This technology utilizes an anchored multiplex polymerase chain reaction (AMP) technique that detects gene rearrangements in a fusion partner agnostic manner. FASTQ data analysis, including fusion calling, was performed by ArcherDx Analysis software v5.0.6 using default parameters. The detailed list of all genes included in Archer® FusionPlex® is shown in Additional file 1.

### *MGMT* promoter methylation

DNA was extracted from frozen tumor tissue and subjected to bisulfite treatment. Two separate methylation-specific PCR reactions were performed, one using primers specific for methylated *MGMT* promoter sequences, and a second using PCR primers specific for unmethylated *MGMT* promoter sequences [8].

### ATRX immunohistochemistry methods

ATRX immunohistochemistry was preformed using ATRX Cat # BSB-3295 from Bio SB. RTU (ready to use) pretreatment ER2 (EDTA ph 9.0) for 15 min. The clone BSB-108 was used, as previously reported [21].

### Statistical analysis

The statistical association of *TERT*p-wt GBM with other factors, including age, sex, other genomic alterations, and location of tumor, were analyzed using the Fisher exact test. The association of *TERT*p-wt GBM with ATRX immunohistochemistry, *MGMT* promoter methylation status, and presence of fusion gene by solid fusion panel were each also evaluated. Cases with unavailable molecular or IHC data were excluded from the final correlation analysis.

The data were analyzed using the Fisher exact test. Description of overall survival (OS) was estimated by the Kaplan-Meier product limit method. A two-tailed *P* value of < 0.05 was considered to be statistically significant.

## Results

### Patient demographic and tumor characteristics

We identified 121 adult GBM cases with available molecular and immunohistochemistry data between 2016 and 2018 (Additional file 2). We excluded histologic GBMs containing *IDH* R132 and *H3F3A* mutations from statistical analyses (*n* = 11 and *n* = 1, respectively), for the reasons noted above [15].

**Fig. 1:**
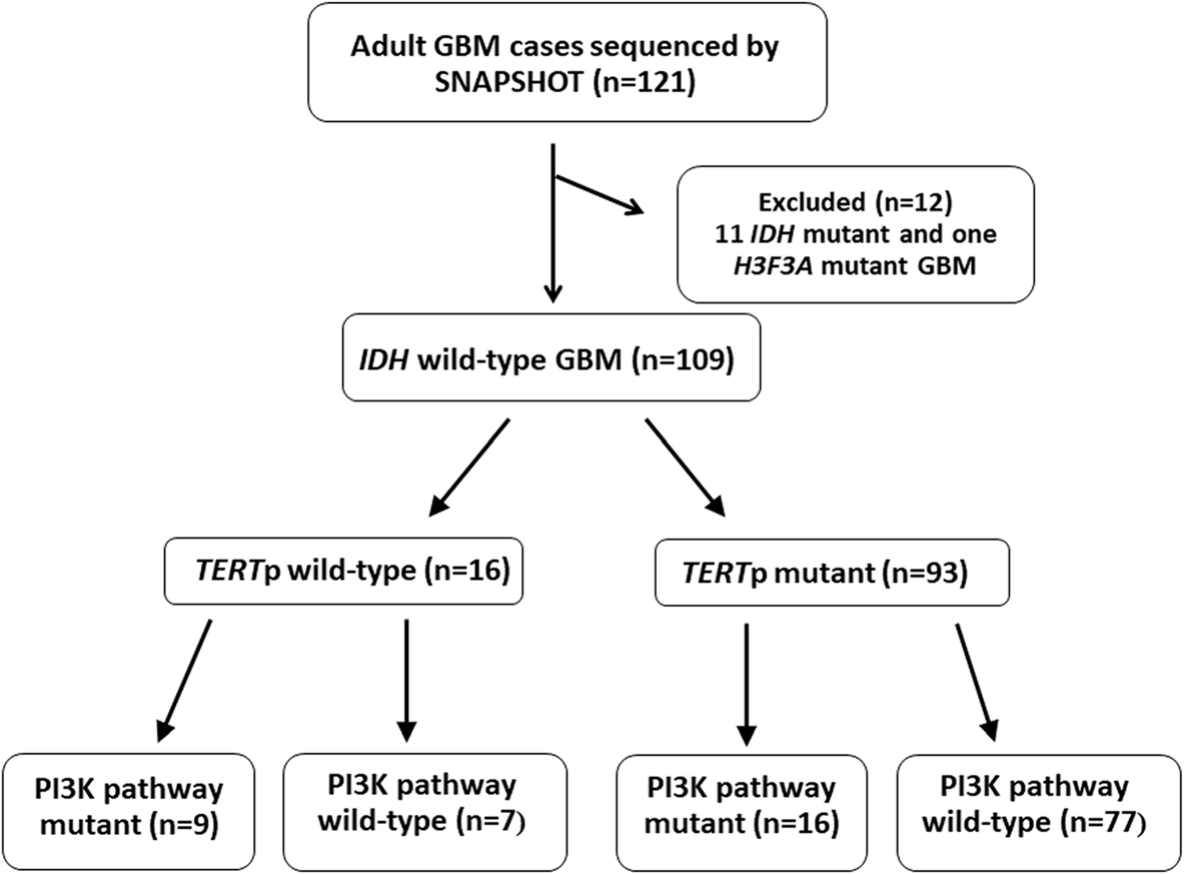
Flowchart depicting mutation breakdown of adult glioblastoma cases

Within this cohort (*n* = 109), the average age of patients was 60 years (range 18–84 years). Genetic alterations in the *TERT* gene were detected in 93 tumors; 92 were *TERT*p mutant (84.4%), and an additional case had a *TERT-SUB* fusion. The remaining 16 patients (14.7%) had *TERT*p-wt GBM (Fig. 1 and Additional file 2). The average age of patients with *TERT*p-wt GBMs was 53.2 years, which was significantly younger than the average age of their counterparts with *TERT*p mutant GBMs (60.7 years, *p* = 0.0096), and significantly older than the average age of patients with *IDH* mutant GBMs (38.6 years, *p* = 0.0041).

Across the cohort of *IDH*-wt GBM, the male to female ratio was 1.66. *TERT*p-wt GBMs did manifest a numerically higher proportion of male patients (13/16, 87.5%), compared with 55/93 male patients (59%) with *TERT*p mutant GBMs, but this difference was not statically significant (*p* = 0.103).

We examined the location of the primary tumor presentation. In the *TERT*p-wt group, the primary tumors were mainly found in a supratentorial (13) and thalamic/midline location (1), but also in a cerebellar site (3 cases). In contrast, in the *TERT*p mutant group, the tumors were exclusively located supratentorially (91) or thalamic/midline (2), with none found in the cerebellum. Consequently, a significant correlation between *TERT*p-wt status and cerebellar location (*p* = 0.0027) was observed. Of note, one of the cerebellar GBMs occurred in a patient with a *NF1* germline mutation (Neurofibromatosis type 1).

The median time of follow-up in surviving patients was 189 days for the *TERT*p-wt group and 246 days for the *TERT*p mutant group. Due to the short follow-up time, survival analyses may be underpowered to detect differences. Nonetheless, no detectable difference in survival was observed between the two groups (*p* = 0.74).

### Genetic and epigenetic correlation

We examined genetic and epigenetic correlations between *TERT*p-wt versus mutant tumors. Four *TERT*p mutant cases were found to harbor a hotspot *BRAF* V600E mutation, which is characteristic of epithelioid GBM [14]. *NF1* mutations were more commonly seen in *TERT*p-wt GBMs (6/16, 37.5%), in comparison with 18/93 (19%) in the *TERT*p mutant GBM cohort, however, this was not a statistically significant difference (*p* = 0.11). Also, we did not observe a significant difference in *MGMT* promoter methylation status in the *TERT*p-wt group vs. the mutant group (7/14 vs. 36/90, *p* = 0.56).

Activating alterations in the PI3K pathway (mainly *PIK3CA* or *PIK3R1*) were detected in 25 out of 109 cases in the cohort (23%) (Additional file 3). Interestingly, we observed a strong correlation between *TERT*p-wt status and mutations targeting the PI3K pathway: 9/16 (56%) of *TERT*p-wt GBMs contained a PI3K pathway alteration, while only 16/93 (17%) of mutant GBMs harbored these alterations (*p* = 0.0018) (Fig. 1). Furthermore, we detected an inverse correlation between *PIK3CA*/*PIK3R1* and *EGFR* alterations. Only 2/25 cases (8%) with a PI3K pathway alteration had an *EGFR* mutation or *EGFRvIII*, whereas 38/82 of PI3K wild-type GBM had an *EGFR* alteration (46.3%, *p* = 0.0003).

**Fig. 2:**
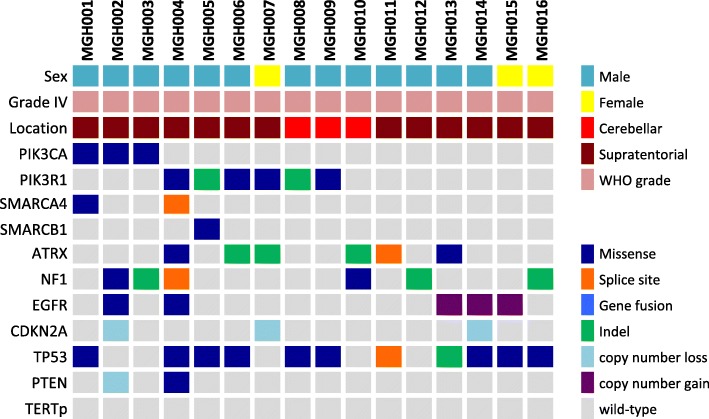
Summary of clinical features and molecular alterations in *TERT*p-wt glioblastoma

Moreover, as expected, *ATRX* mutations were detected by sequencing in 6/16 (37.5%) *TERT*p-wt GBMs, while only 6/93 (6.5%) of *TERT*p mutant GBMs had an *ATRX* mutation. Consequently, this manifested as a significant correlation between *TERT*p-wt status and *ATRX* mutation (*p* = 0.0022). Of note, our workflow for assigning mutation was highly sensitive, leading to potential false positive assignments of *ATRX* candidate alterations that may not functionally inactivate the protein product. The further assessment of ATRX loss-of-expression using immunohistochemistry revealed a similarly significant result: 4/13 (31%) of *TERT*p-wt GBMs had ATRX loss vs. 0/80 mutant GBMs (*p* = 0.0002) (Fig. 2).

**Fig. 3:**
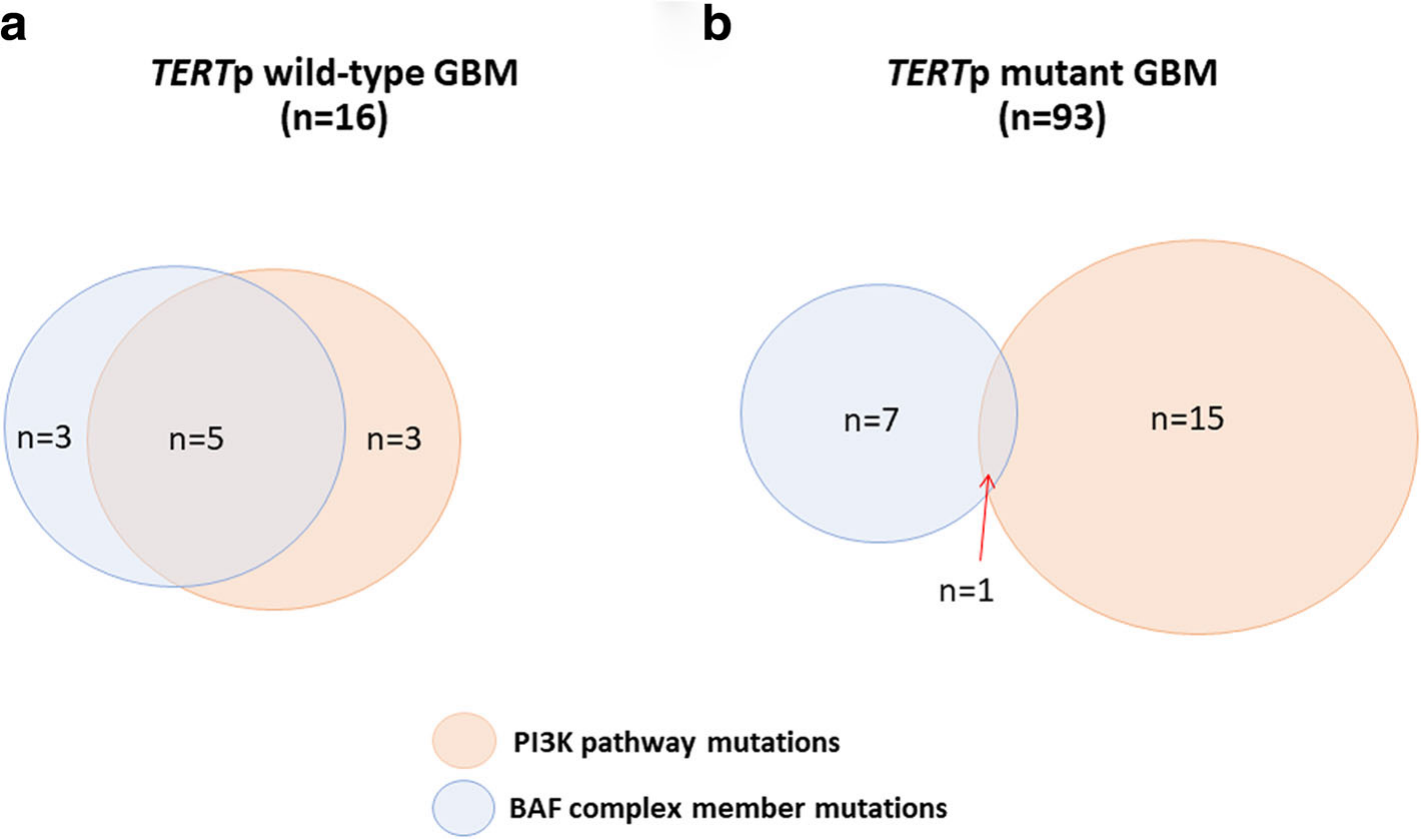
Venn diagram depicting BAF complex mutation (*SMARCA4, SMARCB1, ATRX, or ARID1A*) and PI3K mutations relationship in *TERT* p-wt (**a**) and *TERT* p mutant (**b**) glioblastomas (GBM). Cases negative for both BAF complex and PI3K mutations amounted to *n* = 5 and *n* = 70, respectively

Finally, we noted that 8/16 (50%) of *TERT*p-wt GBMs harbored mutations in the BAF complex gene family (*SMARCA4*, *SMARCB1*, *ATRX*, and *ARID1A*), compared with only 8/93 of *TERT*p mutant GBMs (*p* = 0.0002). Given the role of *ATRX* in telomere maintenance, mutations in either group (ATRX vs SWI/SNF) may be unrelated. Nevertheless, we found that this association remained significant when excluding *ATRX* (3/16 (18.8%) of *TERT*p-wt GBMs harboring mutations compared with only 2/93 of *TERT*p mutant GBMs, *p* = 0.022). When combined with our analyses above, we detected a significant difference in co-occurrence between mutations in the BAF complex and PI3K pathway genes by comparing the *TERT*p-wt (*n* = 5/16) and *TERT*p mutant groups (*n* = 1/93, *p* = 0.0002) (Fig. 3).

## Discussion

The WHO 2016 established an *IDH* wild-type subgroup of GBM, comprising the majority of adult grade IV gliomas, yet, this diagnostic grouping still contains significant heterogeneity. In an effort to better sub-classify *IDH*-wt GBMs, we used a broad panel of genes to genotype a large cohort of these neoplasms. In our analyses, we show that the *TERT*p-wt subgroup of *IDH*-wt GBM contains a distinct clinical and molecular profile.

Our findings should be interpreted in the context of extensive recent work studying adult high-grade gliomas. Over the last several years, a strong relationship has been demonstrated between mutational status and clinical, radiological, and molecular characteristics in adult diffuse gliomas [1, 2, 7]. Recently, Eckel-Passow et al. identified five main glioma molecular groups based on three alterations: 1p/19q co-deletion, and *TERT*p and *IDH* mutations. The groups had different ages of onset, survival, and associations with germline variants [7]. In addition, Aibaidula et al. specifically examined the adult *IDH* wild-type lower-grade gliomas, demonstrating significant heterogeneity within this group, with differences in prognosis based on further molecular classification by biomarkers such as *TERT*p mutation, *EGFR* amplification, *H3F3A* mutation, and *MYB* amplification [1]. Focusing on GBM, Arita et al. highlighted the importance of *TERT*p mutation, *IDH* mutation, and *MGMT* promoter methylation status on prognosis [2]. Furthermore, Stichel et al. demonstrated the potential of *EGFR* amplification, combined chromosome 7 gain and chromosome 10 loss, and *TERT*p mutations for classifying *IDH* wild-type GBM [24].

*TERT*p mutant GBMs show increased telomerase activation due to the increased TERT expression. In comparison, it is well-established that *IDH* mutant astrocytic gliomas often display the characteristic phenotype termed “alternative lengthening of telomeres” or ALT, associated with mutations in *ATRX* [10, 11, 13]. Another study that attempted to further sub-classify the 5 integrated WHO glioma groups by *ATRX* and *TERT* promoter status showed that *ATRX* alterations were enriched in *TERT*p-wt GBM [19]. A further study of the *TERT*p-wt subgroup by Diplas et al. identified *SMARCAL1* as an additional mechanism of telomere maintenance within this subgroup [6].

In agreement with prior studies, we observed that *TERT*p-wt patients are significantly younger than their *TERT*p mutant counterparts [7] (Additional file 4). In addition, we identified a significantly higher rate of cerebellar GBM in the *TERT*p-wt compared with the *TERT*p mutant patients. Our finding is consistent with prior studies that have shown that cerebellar GBMs occur in patients that are younger than patients with supratentorial GBMs, and have decreased frequency in *TERT*p mutations and more frequent *NF1* mutations [16, 20]. Taken together with our findings, these data support the proposal that cerebellar GBMs may comprise a distinct subclass of tumor, which may arise via an alternative molecular etiology when compared to supratentorial *TERT*p mutant GBM.

PI3K pathway alterations are frequently detected in gliomas, most commonly in grade IV lesions [4, 9]. Our data demonstrate that *TERT*p-wt GBMs are significantly enriched for PI3K pathway mutations compared with *TERT*p mutant GBM. Moreover, mutations in *ARID1A* and other components of the SWI/SNF chromatin remodeling complex (collectively known as the BAF complex), have been previously reported to be frequent in various cancer types (e.g. endometriosis-associated ovarian cancers, endometrial cancers and non-gynecological tumors) [3, 5, 22, 23]. Interestingly, in these cancers, alterations of gene encoding for components of the BAF complex frequently co-occur with activating mutations in *PIK3CA* [3, 23]. It has been additionally reported that dysregulation of the PI3K signaling pathway and loss of function of *ARID1A* may have a combination effect on tumor development [5, 22]. We speculate that this association may extend to a specific subset of gliomas, namely *TERT*p-wt GBM cases, which we find are enriched for BAF complex alterations and activating mutations in genes within the PI3K pathway. Following the logic of WHO 2016 classification, our findings suggest the potential definition of a molecular subtype of high-grade glioma, with implications for the utilization of targeted therapy in these patients [22].

## Conclusions

In conclusion, this study identifies frequent PI3K pathway and BAF complex genetic alterations as co-occurring hallmarks of *TERT*p-wt GBM, potentially reflecting a unique molecular etiology of these tumors. If further validated, these findings may have significant implications for the sub-classification of *IDH*-wt GBM. Optimal management of these patients remains to be defined, but at a minimum, our data suggest that *TERT*p-mutant and *TERT*p-wt GBMs should be analyzed separately in future clinical studies, as they likely comprise distinct subclasses of neoplastic disease.

## Additional files

Additional file 1: A detailed list of all genes included in the SNaPshot v2 panel. (DOCX 14 kb)

Additional file 2: A table including patients’ and tumor characteristics. (XLSX 20 kb)

Additional file 3: A table listing all detected PI3K alterations in the cohort. The majority of alterations were reported in COSMIC (https://cancer.sanger.ac.uk/cosmic/browse/genome) and/or occured at hospot locations in TumorPortal (http://www.tumorportal.org/). Reference human transcripts used: ENST00000263967.3 (PIK3CA) and ENST00000521381.1 (PIK3R1). (XLSX 11 kb)

Additional file 4: Age distribution according to TERTp mutations. (TIF 462 kb)
