# Supplementary material for: TERT promoter wild-type glioblastomas show distinct clinical features and frequent PI3K pathway mutations
Source: Acta Neuropathol Commun. 2018 Oct 17;6:106. doi: 10.1186/s40478-018-0613-2 (PMC6193287; doi:10.1186/s40478-018-0613-2)
Supplement: Supplementary file 1 — A detailed list of all genes included in the SNaPshot v2 panel. (DOCX 14 kb) [file 40478_2018_613_MOESM1_ESM.docx]

**Supplemental data:**

Genes included on the SNaPshotv2 panel (108 genes):

ABL1 (4-7), AKT1 (3,6), ALK (21-23,25), APC (16), ARID1A (1-20), ATM (1-63), ATRX (1-35), AURKA (2,5-8), BRAF (11,15), BRCA1 (2-23), BRCA2 (2-27), CCNE1 (3-8,10,12), CDH1 (1-16), CDK4 (2-7), CDKN2A (1-3), CIC (1-20), CSF1R (7,22), CTNNB1 (3), DAXX (1-8), DDR2 (12-18), DDX3X (1-17), EGFR (3,7,15,18-21), ERBB2 (8,10,19-21,24), ERBB3 (2-3,7-8), ERBB4 (3-4,6-9,15,23), ESR1 (8), EZH2 (16), FBXW7 (1-11), FGFR1 (4,7-8,13,15,17), FGFR2 (7,9,12,14), FGFR3 (7-9,14-16,18), FLT3 (11,14,16,20), FOXL2 (1), GNA11 (5), GNAQ (4-5), GNAS (6-9), H3F3A (2), HNF1A (3-4), HRAS (2-3), IDH1 (3-4), IDH2 (4), JAK2 (11,13-14,16,19), JAK3 (4,13,16), KDR (6-7,11,19,21,26-27,30), KEAP1 (2-6), KIT (2,8-11,13-15,17-18), KRAS (2-5), MAP2K1 (2,3,6-7), MAP3K1 (1-20), MDM2 (2-4,6,8,10), MEN1 (2-10), MET (2,11,14,16,19,21), MLH1 (12), MPL (10), MSH6 (1-10), MSI, MYC (1-3), MYCN (3), NF1 (1-58), NF2 (1-15), NOTCH1 (25-27,34), NPM1 (11), NRAS (2-5), PIK3CA (2,5,7-8,10,14,19,21), PIK3R1 (1-10), POLE (9-14), PTCH1 (1-23), PTEN (1-9), PTPN11 (3,13), RB1 (1-27), RET (10-11,13-16), RHOA (2-3), RNF43 (2-10), ROS1 (38), SDHB (1-8), SMAD2 (7), SMAD4 (2-12), SMARCA4 (3-36), SMARCB1 (2,4,5,9), SMO (3,5-6,9,11), SRC (14), STAG2 (3-34), STK11 (1-9), SUFU (1-12), TERT (1), TP53 (1-11), TP63 (1-14), TSC1 (3-23), TSC2 (2-42), TSHR (10), VHL (1-3)

Archer® FusionPlex® Solid Tumor (AK0034) kit validated fusion targets:

ALK (19-22, intron 19), BRAF (7-12, 15), BRD4 (10, 11), EGFR (2-7 exon skipping/vIII variant, 7-9, 16, 20, 24, 25), EWSR1 (4-14), FGFR2 (2, 8-10, 17), MAML2 (2, 3), MET (exon 14 skipping), NRG1 (1-3, 6), NUTM1 (3), RET (8-13), and ROS1 (31-37). Additional targeted genes (exons) include: AKT3 (1-3), AR (1-8), ARHGAP26 (2, 10-12), AXL (19,20), BRD3 (9-12), CSF1 (5-9), CSF1R (7,11-13,22), ERG (2-11), ESR1 (3-6), ETV1 (3-13), ETV4 (2, 4-10), ETV5 (2, 3, 7-9), ETV6 (1-7), FGFR1 (2, 8-10, 17), FGFR3 (8-10, 17, intron 17), FGR (2), INSR (12-22), JAZF1 (2-4), MAST1 (7-9, 18-21), MAST2 (2, 3, 5, 6), MET (13, 15), MSMB (2-4), MUSK (7-9, 11-14), MYB (7-9, 11-16), NOTCH1 (2, 4, 26-31, internal exon 3-27 deletion), NOTCH2 (5-7, 26-28), NTRK1 (8,10-13), NTRK2 (11-17), NTRK3 (13-16), NUMBL (3), PDGFRA (7, exon 8 deletion, 10-14), PDGFRB (8-14), PIK3CA (2), PKN1 (10-13), PPARG (1-3), PRKCA (4-6), PRKCB (3), RAF1 (4-7, 9-12), RELA (3, 4), RSPO2 (1, 2), RSPO3 (2), TERT (2), TFE3 (2-8), TFEB (1,2), THADA (24-31,36), and TMPRSS2 (1-6).
