# Supplementary figures and images for: TERT promoter wild-type glioblastomas show distinct clinical features and frequent PI3K pathway mutations
Source: Acta Neuropathol Commun. 2018 Oct 17;6:106. doi: 10.1186/s40478-018-0613-2 (PMC6193287; doi:10.1186/s40478-018-0613-2)

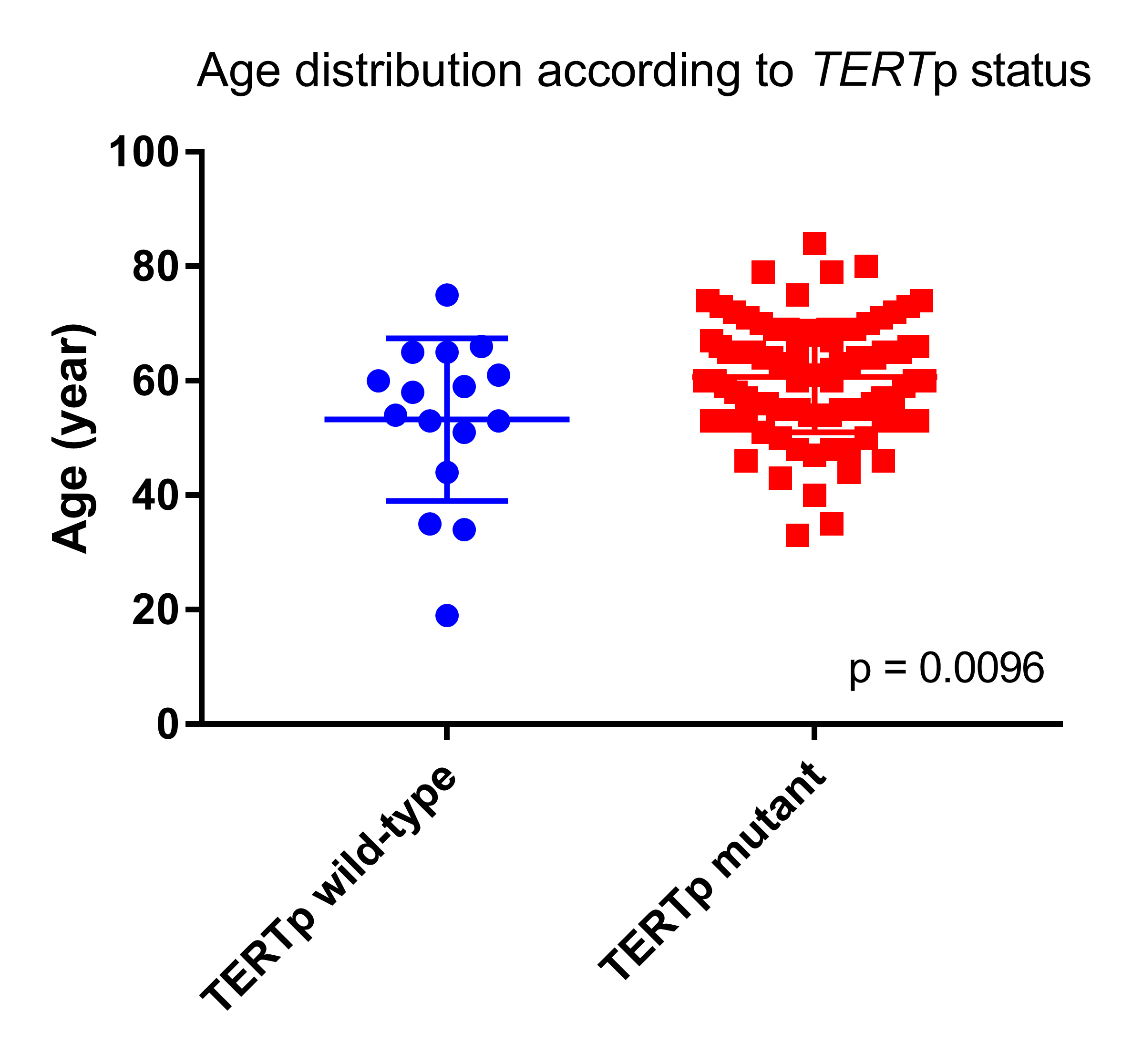

Supplement: Supplementary file 4 — Age distribution according to TERTp mutations. (TIF 462 kb) [file 40478_2018_613_MOESM4_ESM.tif]
